# Biochemical Evaluation of the Effects of Hydroxyurea in Vitro on Red Blood Cells

**DOI:** 10.3390/antiox10101599

**Published:** 2021-10-12

**Authors:** Cristiane Oliveira Renó, Grazielle Aparecida Silva Maia, Leilismara Sousa Nogueira, Melina de Barros Pinheiro, Danyelle Romana Alves Rios, Vanessa Faria Cortes, Leandro Augusto de Oliveira Barbosa, Hérica de Lima Santos

**Affiliations:** 1 Laboratório de Bioquímica Celular, Campus Centro Oeste Dona Lindu, Universidade Federal de São João del-Rei, Divinópolis 35501-296, Minas Gerais, Brazil; cristianereno@outlook.pt (C.O.R.); graquimica@ufsj.edu.br (G.A.S.M.); leilismara@ufsj.edu.br (L.S.N.); cortesvf@ufsj.edu.br (V.F.C.); lbarbosa@ufsj.edu.br (L.A.d.O.B.); 2 Laboratório de Análises Clínicas, Campus Centro Oeste Dona Lindu, Universidade Federal de São João del-Rei, Divinópolis 35501-296, Minas Gerais, Brazil; melinapinheiro@ufsj.edu.br; 3 Laboratório de Hematologia Clínica, Campus Centro Oeste Dona Lindu, Universidade Federal de São João del-Rei, Divinópolis 35501-296, Minas Gerais, Brazil; danyelleromana@ufsj.edu.br

**Keywords:** hydroxyurea, red blood cells, energy metabolism, antioxidant system, reactive oxygen species

## Abstract

Hydroxyurea (HU) is a low-cost, low-toxicity drug that is often used in diseases, such as sickle cell anemia and different types of cancer. Its effects on the red blood cells (RBC) are still not fully understood. The in vitro effects of HU were evaluated on the biochemical parameters of the RBC from healthy individuals that were treated with 0.6 mM or 0.8 mM HU for 30 min and 1 h. After 30 min, there was a significant increase in almost all of the parameters analyzed in the two concentrations of HU, except for the pyruvate kinase (PK) activity. A treatment with 0.8 mM HU for 1 h resulted in a reduction of the levels of lipid peroxidation, Fe^3+^, and in the activities of some of the enzymes, such as glutathione reductase (GR), glucose-6-phosphate dehydrogenase (G6PD), and PK. After the incubation for 1 h, the levels of H_2_O_2_, lipid peroxidation, reduced glutathione (GSH), enzymatic activity (hexokinase, G6PD, and superoxide dismutase (SOD) were reduced with the treatment of 0.8 mM HU when compared with 0.6 mM. The results have suggested that a treatment with HU at a concentration of 0.8 mM seemed to be more efficient in protecting against the free radicals, as well as in treating diseases, such as sickle cell anemia. HU appears to preferentially stimulate the pentose pathway over the glycolytic pathway. Although this study was carried out with the RBC from healthy individuals, the changes described in this study may help to elucidate the mechanisms of action of HU when administered for therapeutic purposes.

## 1. Introduction

Hydroxyurea (HU) is the main drug of choice for the treatment of sickle cell anemia (SCA) and it provides therapeutic benefits through its multiple mechanisms of action. It is easily synthesized by urea hydroxylation and acts by inhibiting an enzyme that is involved in the synthesis of ribonucleic acid. In addition to HU being used to treat hematological diseases, such as chronic myeloid leukemia, and its ability to generate nitric oxide, a potent vasodilator, also helps to relieve the patients’ pain [1,2,3]. HU acts on the bone marrow and due to its cytotoxic effects, it decreases the production of blood cells. Furthermore, it stimulates the increase in the synthesis of HbF, which contributes to the reduction of painful crises and vaso-occlusive processes [2].

The drug is administered in different doses, which differ according to the patient and the disease to be treated. In SCA, the doses range from 15–35 mg/kg/day, and in resistant chronic myeloid leukemia (CML) from 1000–2000 mg/day. This drug is inexpensive. It is administered as a single agent and orally. Based on currently available data, the HU treatment fulfills the criteria established for SCA and it should be offered much more frequently [1,2,4]. The absorption of HU is fast and it occurs in the intestine, with wide body distribution [2,5,6] and maximum plasma doses, ranging between 0.2 mM and 0.8 mM, depending on the dose to which the patient is submitted [7].

Despite being widely used for many years, the effects of HU on human RBC have not been fully clarified. Some studies have linked the use of HU to the oxidation of hemoglobin, with increased lipid peroxidation in the membrane of these cells [8,9].

The main physiological role of the RBC is the transport of gases (O_2_, CO_2_) from the lungs to the tissues and vice versa, in addition to maintaining the systemic acid/base balance. They have important antioxidant systems, which contribute substantially to their functions and integrity. The damage of RBC integrity, as well as to their membranes, has been shown to contribute significantly to serious pathologies [10,11,12].

Owing to the importance of the RBC, particularly due to their great carrier potential for drug delivery, it is necessary to broaden the understanding of the HU actions in these cells under non-pathological conditions. This knowledge may be applied to diseases in which HU is included in pharmacotherapeutic protocols. In this context, the authors have aimed to describe the effects of HU in the RBC by evaluating some aspects that are related to the membrane profile, the energy metabolism, and the antioxidant system.

## 2. Materials and Methods

### 2.1. Ethics Statement

The present research project was approved by the Ethics Committee on Human Research of the Federal University of São João del Rei, Brazil (n° 2.977.566). All of the enrolled subjects signed an informative consent form.

### 2.2. Study Samples

This study included six volunteer and healthy adults aged between 24 and 35 years old, of both genders, who were selected from the community of the Federal University of São João del-Rei, Brazil. As the aim has been to evaluate the effect of hydroxyurea on healthy erythrocytes and not characterize its biological effect, the number of samples was small, but this is common practice in similar experiments [13,14,15]. All of the participants were healthy. This was confirmed by self-reported normal blood tests and an absence of hemoglobin S, in addition to no clinical symptoms. The exclusion criteria were a personal, or a family history of vascular disease, coagulopathies, anemia, the use of daily medications, or current or recent pregnancy. Thirty mL venous blood samples were collected after 8–12 h of fasting when using EDTA as an anticoagulant and were transported between 2 and 8 °C. These were equally distributed into three groups, including (1) control (with no HU), (2) addition of 0.6 M HU, and (3) addition of 0.8 M HU. All of the samples were analyzed at 30 and 60 min after adding HU. The concentrations chosen above were based on the therapeutic regimen of HU, which was adopted for patients with vaso-occlusive crises, that is, 15–35 mg/kg and a plasma peak of between 0.2–0.8 mM [7]. During the incubation period, the samples were kept at 4 °C and after this, the red blood cells concentrate was separated by centrifugation at 700× *g* and at 4 °C for 10 min when using a CT18000R (Cientec^®^) centrifuge (Belo Horizonte, MG, Brazil). The supernatant of each sample was removed, aliquoted, and stored in a freezer at −20 °C until required.

### 2.3. Preparation of Hemolysate

All of the RBC concentrates were subjected to three successive washes, then doubling the initial sample volume with 0.9% NaCl solution, and centrifuging for 10 min at 700× *g*. After the last wash, each pellet of RBC was lysed, in the proportion of 1:20 when using a hemolysis solution containing 0.27 M EDTA and 0.007 mM β mercaptoethanol. This was then frozen in acetone in a freezer at −20 °C. The hemolysate was thawed at room temperature and kept on ice for further enzymatic analyses.

### 2.4. Determination of the Hemoglobin (Hb) Concentration

The determination of the Hb concentration in the hemolysates and plasma was performed by the Cyanomethemoglobin Method, which was adapted from Dacie and Lewis [16], accessed on 6 March 2019 when using Drabkin’s solution (0.9 mM potassium ferricyanide and 1.5 mM potassium cyanide), as well as a hemoglobin standard of known concentration (10 g/dL). The reading was performed by scanning on a GENESYS™ 10 UV/Vis spectrophotometer (Thermo Scientific^®^, Waltham, MA, USA), at 540 nm for 5 min. The Hb concentrations of the samples were calculated by using the calibration factor that was obtained by the absorbance of the Hb standard.

### 2.5. Oxidative Stress Indicators

#### 2.5.1. Lipid Peroxidation

To investigate lipid peroxidation, the plasma levels of the thiobarbituric acid reactive substances (TBARS) were determined. For this, 100 µL of plasma was added to a tube containing 1 mL of a solution that was made with 15% trichloroacetic acid, 0.38% thiobarbituric acid, and 0.25 N hydrochloric acid. The mixture was incubated at 100 °C for 30 min and then centrifuged at 3000 rpm for 10 min. The optical density (OD) was measured at 535 nm when using a GENESYS™ S10 UV/Vis spectrophotometer (Thermo Scientific^®^, Waltham, MA, USA). The malondialdehyde (MDA) content was determined by using a standard curve that was constructed in a concentration range of 1 to 100 nM [17,18].

#### 2.5.2. Determination of Fe^3+^ in the Residual Plasma Samples

The Fe^3+^ quantification was performed as described by Adams [19], with slight modifications. In a test tube, 150 μL of plasma was mixed with 1.5 mL of 1 M KSCN, and the volume was adjusted to 3.0 mL with 0.9% NaCl. The samples were then homogenized using Vortex Multifuncional K40-1020 (KASVI^®^, São José dos Pinhais, PR, Brazil), and the optical density was read at 480 nm (OD480) by using GENESYS™ 10 UV/Vis spectrophotometer (Thermo Scientific^®^, Waltham, MA, USA). A standard curve was constructed when using a 1 M FeCl_3_ solution as a standard. The OD was stable for 15 min.

#### 2.5.3. Quantification of Hydrogen Peroxide (H_2_O_2_)

The quantification of the H_2_O_2_ production was performed by adding 20 μL of hemolysate to a reaction medium containing 250 μM of ammoniacal ferrous sulfate, 25 mM of H_2_SO_4_, and 100 μM of xylenol orange in a 1 mL Eppendorf^®^ tube. After vortexing for 10 s, the tube was at rest and protected from the light for 45 min, and then the product formed was read on a GENESYS™ 10 UV/Vis scanning spectrophotometer (Thermo Scientific^®^, Waltham, MA, USA) at a wavelength of 580 nm [20]. The production of H_2_O_2_ was calculated from a standard curve by using hydrogen peroxide pure (H_2_O_2_) in concentrations from 0 to 100 nM.

#### 2.5.4. Determination of the Superoxide Dismutase (SOD) Activity

The SOD activity was determined through a method that was adopted in the authors’ laboratory when using epinephrine in an alkaline medium since it is converted into adrenochrome, producing O^.−2^, which is a SOD substrate. The SOD activity was defined by assessing its ability to inhibit epinephrine oxidation. The 100% oxidation was defined in a tube containing a medium, with glycine (50 mM, pH 10) and 25 µL of epinephrine (60 mM pH = 2). The other tubes contained the same medium but with different volumes of hemolysate (2–6 μL). The oxidation reaction was measured and the OD of each condition was read at 480 nm on a GENESYS™ 10 UV/Vis scanning spectrophotometer (Thermo Scientific^®^, Waltham, MA, USA). The enzyme activity was expressed in units required to inhibit 50% of the rate of the adrenochrome formation [21].

#### 2.5.5. Determination of the Catalase Activity

The catalase activity was determined in RBC hemolysates and was based on the method of Aebi [22], with some modifications. Immediately before determining the activity of this enzyme, the hemolysate was again centrifuged and the supernatant was used for this purpose. Twenty (20) μL of this hemolysate supernatant was added to a cuvette containing 2 mL of 50 mM phosphate buffer (pH 7.0). The reactions were initiated by the addition of 3.4 μL of freshly prepared 30% H_2_O_2_. The decomposition of H_2_O_2_ was followed spectrophotometrically at 240 nm, using a GENESYS™ 10 UV/Vis scanning spectrophotometer (Thermo Scientific^®^, Waltham, MA, USA). The activity was estimated from the slope and expressed as micromoles of H_2_O_2_ decomposed per minute.

#### 2.5.6. Determination of the Enzymatic Activity of Glutathione Peroxidase (GPx)

The GPx activity was determined by following the oxidation of NADPH when using hydrogen peroxide at a wavelength of 340 nm. The Flohe and collaborators [23] and the Nakamura and Hosada [24] methods were adapted by using a reaction medium, with a 50 mM phosphate buffer, 4 mM EDTA, 1 mM GSH, 1.25 mM NaN_3_, 0.16 mM NADPH, and 5 µL of hemolysate. The total volume was adjusted to 1 mL with water. The reaction was initiated by the addition of 4 mM H_2_O_2_. Modifications in the OD at 340 nm were observed within 2 min. The enzyme activity was measured at nmol NADPH·min^−1^·L/gHb [23,24].

#### 2.5.7. Determination of the Content of Reduced Glutathione (GSH)

The GSH concentration was evaluated by adding 5 µL of hemolysate to a reaction medium containing 0.1 M phosphate buffer (pH 8.0) and DNTB/EDTA 10 mM. After 15 min at room temperature, the solution was read by a GENESYS™ 10 UV/Vis scanning spectrophotometer (Thermo Scientific^®^, Waltham, MA, USA) at a wavelength of 412 nm. The GSH concentration was measured by performing a standard curve (correction factor) at nmolGSH·L/gHb [25,26].

#### 2.5.8. Determination of the Glutathione Reductase (GR) Activity

The GR activity was determined by following the method as described by Racker and collaborators [27], with adaptations. The reaction medium that was used contained a phosphate buffer at pH 7.6, 50 mM EDTA, 0.4 mM, 0.1 mM NADPH, 50 µL of hemolysate, and 1 mM glutathione disulfide (GSSG). The volume was completed with distilled water to 1 mL, and the absorbance of the mixture was read by a GENESYS™ 10 UV/Vis scanning spectrophotometer (Thermo Scientific^®^, Waltham, MA, USA) at a wavelength of 340 nm for 2 min. The enzyme activity was measured at ρmol NADPH·min^−1^·L/gHb [27].

### 2.6. Energy Metabolism of the RBC

#### 2.6.1. Determination of the Glucose-6-Phosphate Dehydrogenase (G6PD) Activity

The G6PD activity was measured by incubating 20 µL of hemolysate at 37 °C for 10 min in a reaction medium containing a buffer (0.1 M Tris-HCl and 0.5 mM EDTA, at pH 8.0), 0.01 M MgCl_2_, 0.2 mM NADP, and 0.6 mM glucose-6-phosphate. The absorbance was measured in a GENESYS™ 10 UV/Vis spectrophotometer (Thermo Scientific^®^, Waltham, MA, USA) at 340 nm, and its variation per minute was evaluated, with and without the substrate (glucose-6-phosphate). The enzyme activity was quantified by reducing NADP^+^ to NADPH. The enzymatic activity was calculated at Ul·gHb^−1^·min^−1^, where U was equal to 1 µmol NADP^+^·min^−1^·mL^−1^ [28].

#### 2.6.2. Determination of the Hexokinase Activity (HEX)

The HEX activity was determined by using 50 µL of hemolysate in a reaction medium containing a buffer (0.1 M Tris-HCl and 0.5 mM EDTA, at pH = 8.0), 0.01 M MgCl_2_, 0.2 mM NADP, 2 mM glucose, 2 mM of ATP, and 0.1 of a unit of glucose-6-phosphate dehydrogenase enzyme. The absorbance was measured in a GENESYS™ 10 UV/Vis spectrophotometer (Thermo Scientific^®^, Waltham, MA, USA) at 340 nm, and its variation per minute was evaluated, with and without ATP. The enzyme activity was quantified by the reduction of NADP^+^ to NADPH. The enzymatic activity was calculated at Ul·gHb^−1^·min^−1^, where U was equal to 1 µmol of NADP^+^·min^−1^·mL^−1^ [28].

#### 2.6.3. Determination of the Pyruvate Kinase (PK) Activity

The PK activity was determined by incubating 20 µL of hemolysate at 37 °C for 10 min in a reaction medium containing a buffer (0.1 M Tris-HCl and 0.5 mM EDTA, at pH = 8.0), 0.01 M MgCl_2_, 0.1 M KCl, 0.2 mM NADH, 1.5 mM ADP, 6 units of lactate dehydrogenase enzyme, and 5 mM phosphoenolpyruvate (PEP). The absorbance was measured in a GENESYS™ 10 UV/Vis spectrophotometer (Thermo Scientific^®^, Waltham, MA, USA) at 340 nm, and its variation per minute was evaluated, with and without PEP. The enzyme activity was quantified by the oxidation of NADPH to NADP^+^. The enzymatic activity was calculated at Ul·gHb^−1^.min^−1^, where U was equal to 1 µmol of NADP^+^·min^−1^·mL^−1^ [28].

### 2.7. Statistical Analysis

The results were analyzed when using GraphPad Prism 5 software. For the analysis of data normality, the Shapiro-Wilk test (for any sample size) was used. For the data with a normal distribution, the Analysis of Variance (ANOVA) was used, followed by Tukey’s multiple comparison test. Values of *p* < 0.05 were considered significant.

## 3. Results

### 3.1. Determination of the Oxidative Stress Indicators

**Figure 1:**
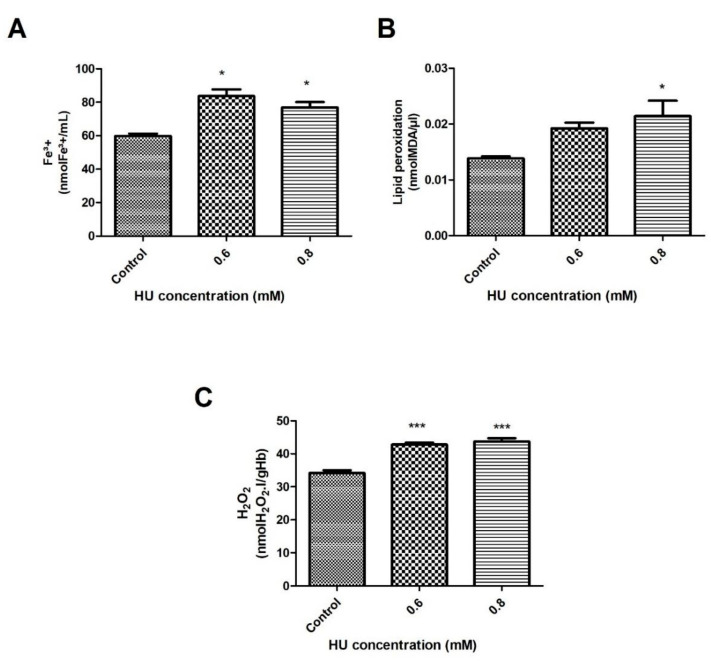
Oxidative stress indicators in the erythrocytes of the control samples that were treated with 0.6 and 0.8 mM HU (*n* = 3) for 30 min. (**A**) Fe^3+^ was measured in plasma; (**B**) Lipid peroxidation (MDA) was measured in plasma; (**C**) Hydrogen peroxide (H_2_O_2_) was measured in hemolysate. In all of the cases, * represents a comparison between the controls and the treated groups (* *p* < 0.05 and *** *p* < 0.001).

Biological markers, such as Fe^3+^, MDA, and H_2_O_2_ are frequently used to predict oxidative stress in the RBC. During a 30-min treatment, it was possible to observe a profile increase of these indicators in the treated groups when compared to the control group, as described below. Figure 1 illustrates that the exposure of the RBC to concentrations of 0.6 and 0.8 mM of HU resulted in an increase in plasma Fe^3+^ of about 39.9% and 28.6%, respectively, compared to the control group (Figure 1A) (*p* < 0.05). Figure 1B depicts the evaluation of lipid peroxidation when using the TBARS method. The MDA content increased by 52% in the HU-treated group at a concentration of 0.8 mM (*p* < 0.05). H_2_O_2_ was also measured because of its ability to react and damage the RBC membrane. When evaluating the total H_2_O_2_ content in the hemolysate, a significant increase of 25% and 28% were found in the concentrations of 0.6 mM and 0.8 mM, respectively (Figure 1C).

**Figure 2:**
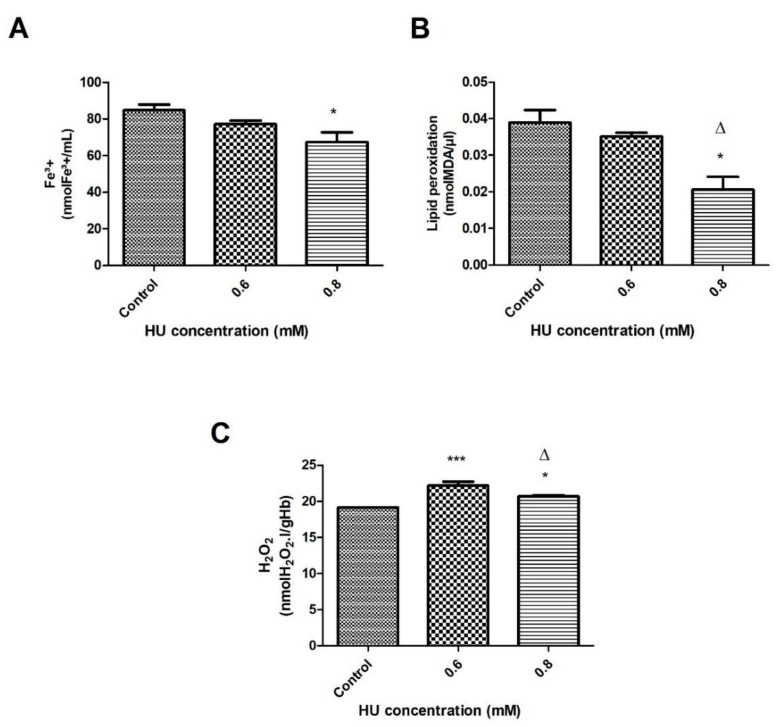
Oxidative stress indicators in the erythrocytes of the control samples that were treated with 0.6 and 0.8 mM HU (*n* = 3) for 1 h. (**A**) Fe^3+^ was measured in plasma; (**B**) Lipid peroxidation (MDA) was measured in plasma; (**C**) Hydrogen peroxide (H_2_O_2_) was measured in hemolysate. In all of the cases, * represents a comparison between the controls and the treated groups (* *p* < 0.05 and *** *p* < 0.001), while ∆ represents a comparison between the groups treated with 0.6 and 0.8 mM HU (*p* < 0.05).

The authors also examined whether the alterations in the oxidative stress indicators were time-dependent. After an hour of incubation with 0.8 mM HU, it was observed that the Fe^3+^ content was 20% lower (*p* < 0.05) when compared with the control groups (Figure 2A). A significant decrease of 47% (*p* < 0.05) in the MDA content in the plasma that was treated with 0.8 mM HU was also found when compared with the control groups. When comparing the groups that were treated with HU ( 60 min), a significant decrease (41%) (*p* < 0.05) in the MDA was observed with the concentration of 0.8 mM HU, in relation to 0.6 mM (Figure 2B).

The hydrogen peroxide content in the hemolysate, as shown in Figure 2C, increased significantly in the treated group at both HU concentrations when compared to control. The increase was 15%, (*p* < 0.001) and 7% at concentrations of 0.6 mM and 0.8 mM (*p* < 0.05), respectively. A significant decrease of 7% (*p* < 0.05) in the H_2_O_2_ was also observed in the sample of hemolysate that was treated with 0.8 mM when compared to that treated with 0.6 mM (Figure 2C).

### 3.2. Antioxidant Defense of the RBC

As reported previously, oxidation is a possible mechanism of damage to the RBC, together with the enzymes of the antioxidant system, such as CAT, SOD, GPx, and GR, which act as protectors of these cells against attack by the reactive species. The possible oxidative effects of HU on the RBC were also measured by the antioxidant enzyme activities in the treated and control samples.

**Figure 3:**
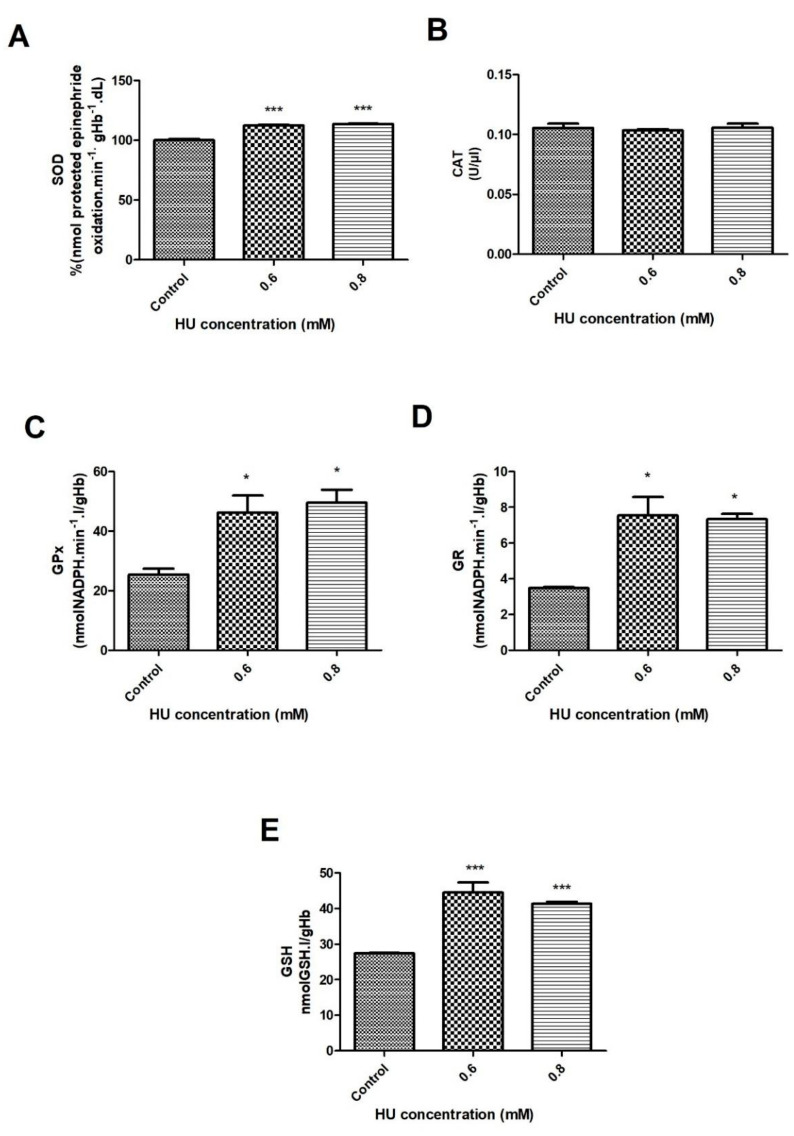
Enzymatic activity of the erythrocyte antioxidant system in the control samples that were treated with 0.6 and 0.8 mM HU (*n* = 3) for 30 min. Hemolysate was used to measure (**A**) Superoxide dismutase (SOD); (**B**) Catalase (CAT); (**C**) Glutathione peroxidase (GPx); (**D**) Glutathione reductase (GR); (**E**) Reduced glutathione. In all of the cases, * it represents a comparison between the control groups and the treated groups (* *p* < 0.05 and *** *p* < 0.001).

**Figure 4:**
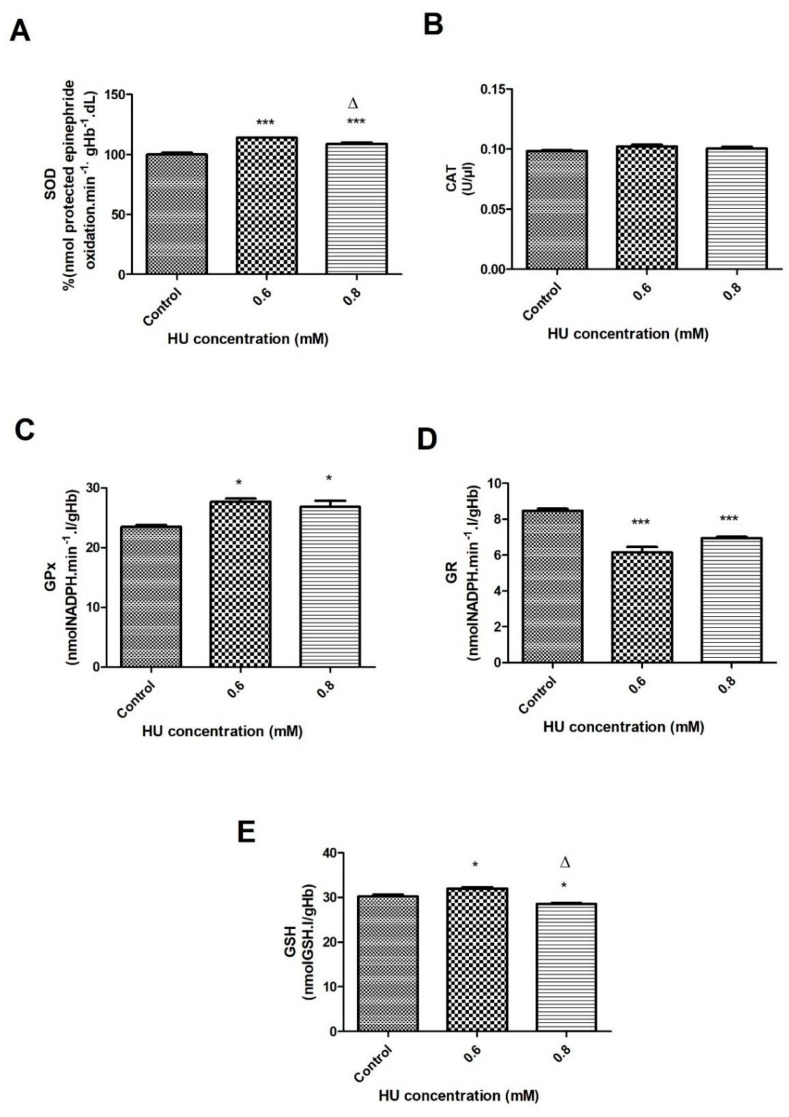
Enzymatic activity of the erythrocyte antioxidant system in the control samples that were treated with 0.6 and 0.8 mM HU (n = 3) for 1 h. Hemolysate was used to measure (**A**) Superoxide dismutase (SOD); (**B**) Catalase (CAT); (**C**) Glutathione peroxidase (GPx); (**D**) Glutathione reductase (GR); (**E**) Reduced glutathione. In all of the cases, * represents a comparison between the controls and the treated groups (* *p* < 0.05 and *** *p* < 0.001), while ∆ represents a comparison between the groups that were treated with 0.6 and 0.8 mM HU (*p* < 0.05).

When evaluating the activity of the SOD enzyme, a significant increase of about 12% was observed after 30 min of incubation with HU, at both concentrations of HU when compared to the control group (*p* < 0.001) (Figure 3A). After 1 h of incubation, it was also possible to observe a significant increase (*p* < 0.001) of 13% and 8%, in the concentrations of 0.6 mM and 0.8 mM of HU, respectively, when compared to the control groups (Figure 4A). When comparing the two treated groups (30 and 60 min), a significant 5% increase occurred in the 0.6 mM HU (Figure 4A).

The catalase activity did not differ significantly among all of the examined groups (Figure 3B and Figure 4B). The activity of the enzyme glutathione peroxidase showed a significant increase at both of the treatment times. The groups that were treated with HU showed an increase of 82% (*p* < 0.05) (30 min) and 15.5% (*p* < 0.05) (1 h) when compared with the control groups (Figure 3C and Figure 4C).

The activity of glutathione reductase in the group that was treated with 0.6 mM HU (after 30 min) showed a significant increase (*p* < 0.05), which was 2 times greater when compared to the control groups (Figure 3D). The 1-h treatment presented a different profile, that is, the samples that were treated with 0.6 and 0.8 mM of HU obtained a significantly lower GR activity (*p* < 0.05) of about 26% and 17%, respectively, compared to the control groups (Figure 4D). The GSH content increased significantly (*p* < 0.05) in the two HU concentrations over 30 min at 62% and 52% in the samples that were treated with 0.6 and 0.8 mM, respectively (Figure 3E). After the 1-h treatment, it was possible to observe a significant increase (*p* < 0.05) of 6% but only at a concentration of 0.6 mM, while a decrease of 6% (*p* < 0.05) was observed in the treated group with 0.8 mM HU when compared to the control groups (Figure 4E). At a concentration of 0.6 mM HU, there was a significant increase of 12% (*p* < 0.05) in the GSH content when compared to the sample that was treated with 0.8 mM HU (Figure 4E).

### 3.3. The Energy Metabolism of the RBC

**Figure 5:**
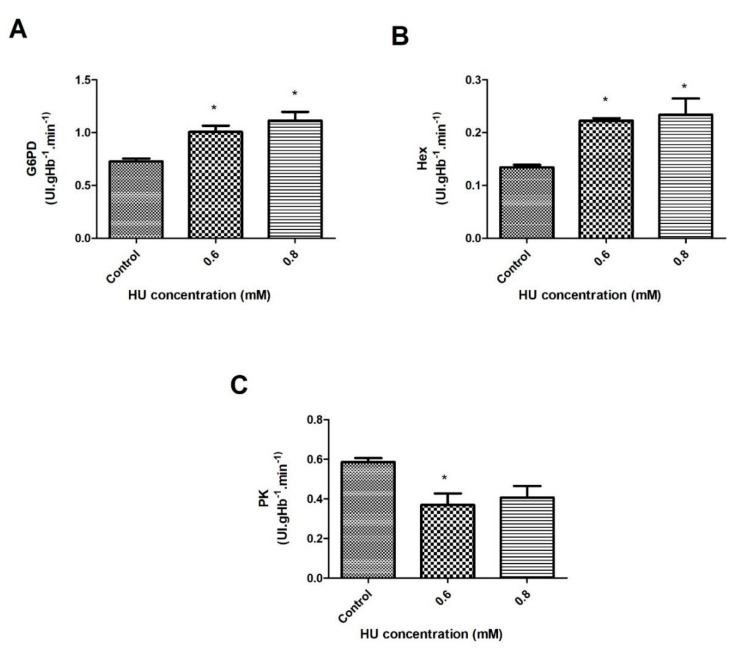
Enzymatic activity of the erythrocyte energy metabolism in the control samples that were treated with 0.6 and 0.8 mM HU (*n* = 3) for 30 min. Hemolysate was used to measure (**A**) Glycose-6-phosphate-dehydrogenase (G6PD); (**B**) Hexokinase (Hex); (**C**) Pyruvate kinase (PK). In all of the cases, * represents a comparison between the control groups and the groups that were treated with HU (* *p* < 0.05).

The pathway (Glucose → Glucose 6-phosphate → 2 ATP + 2 lactate generation) uncouples the ATP generation from the oxygen consumption and it is the only source of metabolic energy for the mature human RBC. The primary outcome revealed that with 30 min of treatment with HU, the G6PD had a significant increase of 37.5% and 54.6% in its activity at concentrations of 0.6 mM and 0.8 mM, respectively (*p* < 0.05), compared to the control groups (Figure 5A).

**Figure 6:**
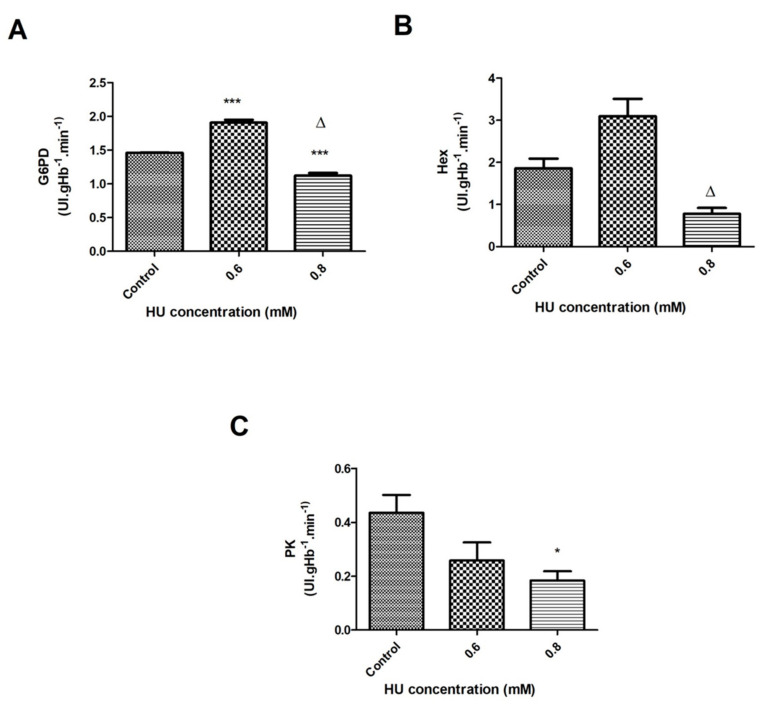
Enzymatic activity of the erythrocyte energy metabolism in the control samples that were treated with 0.6 and 0.8 mM HU (*n* = 3) for 1 h. Hemolysate was used to measure (**A**) Glucose-6-phosphate-dehydrogenase (G6PD); (**B**) Hexokinase (Hex); (**C**) Pyruvate kinase (PK). In all of the cases, * represents a comparison between the control groups and the groups that were treated with HU (* *p* < 0.05 and *** *p* < 0.001), while ∆ represents a comparison between the groups that were treated with 0.6 and 0.8 mM HU (*p* < 0.05).

For the 1-h treatment, a significant increase in the G6PD activity of 30% (*p* < 0.001) was observed at a concentration of 0.6 mM HU, whereas at 0.8 mM of HU, the G6PD activity was lower by 23% (*p* < 0.001), compared to the control groups. When analyzing both of the HU concentrations, the G6PD activity was 42% lower when comparing 0.8 mM to 0.6 mM of HU (Figure 6A).

The hexokinase activity in the 30-min treatment had a significant increase of around 73% at both of the HU concentrations when compared to the control groups (Figure 5B). During the 1-h treatment, it was not possible to observe significant differences between the control groups and the treated groups. There was a significant decrease of about 77% (*p* < 0.05) in the hexokinase values of the sample that was treated with 0.8 mM, compared to that treated with 0.6 mM HU (Figure 6B).

Regarding the activity of pyruvate kinase (PK), it was possible to observe a significant reduction in the PK activity of about 23% (*p* < 0.05) after 30 min of incubation with 0.6 mM HU when compared to the control groups (Figure 5C). Within 1 h, a significant decrease of about 59% (*p* < 0.05) in the PK activity was also observed at a concentration of 0.8 mM, compared with the control groups (Figure 6C). To facilitate a data comparison, the results are summarized in Table 1 and Table 2.

## 4. Discussion

Hydroxyurea is a drug that is used in the treatment of various diseases, such as cancer and sickle cell anemia, in addition to having a high potential to treat other diseases [29]. In the present in vitro study carried out with the RBC from healthy individuals, changes in these cells were observed after incubations of 0.6 mM and 0.8 mM HU for 30 and 60 min.

The results showed that after 30 min of treatment with HU, at both of the concentrations, there was an increase in the Fe^3+^, MDA, and H_2_O_2_ levels (Figure 1A–C, respectively). According to Iyamu and collaborators [9], after treating the blood in vitro with different concentrations of HU, there was an increase in the oxidation of hemoglobin A and hemoglobin S, the latter of which is present in the patients with sickle cell anemia. The oxidation of hemoglobin leads to the formation of methemoglobin; consequently to the release of Fe^3+^, contributing to its increase in the plasma [30]. Fe^3+^ can increase the generation of reactive oxygen species, as well as reacting with the cell membranes, resulting in an increase in lipid peroxidation in both of the cases [31].

MDA is a by-product of the lipid peroxidation of unsaturated phospholipid chains [32], and this increased significantly after 30 min of treatment with 0.8 mM HU, (Figure 1B). In a previous study that was carried out on Wistar rats, which were treated with different doses of HU, the authors reported an increase in the MDA levels that were caused by the drug, proportional to its concentration [29].

Concerning the H_2_O_2_ content, a significant increase in the treated groups was observed, as seen in Figure 1C and Figure 2C. Consequently, this can cause the production of other free radicals, such as the hydroxyl radical (OH^.^). OH^.^, when reacting with Fe^3+^, is one of the most potent oxidants known, and it can accentuate the oxidative stress event [30,33]. Huang and collaborators [34], when performing a treatment in yeasts with different concentrations of HU, observed that there was an increase in the levels of the reactive oxygen species (ROS), especially H_2_O_2_. One hypothesis to explain the increase in the concentration of H_2_O_2_ consists of an increase in the activity of the superoxide dismutase enzyme (SOD), as was observed in the present study (Figure 3A and Figure 4). This enzyme, which is present in the erythrocyte antioxidant system, was responsible for generating H_2_O_2_ by dismuting the superoxide anion to H_2_O_2_.

It has already been described that HU also exhibits a greater oxidative potential of hemoglobin when compared to hydrogen peroxide, whilst under the same conditions of drug concentration and time of exposure, as in this current study [9]. This fact reinforces the hypothesis that HU not only induces an increase in H_2_O_2_ but it is also capable of inducing the formation of other radicals, contributing to a higher oxidation rate.

After 1 h of treatment with 0.8 mM HU, the levels of some oxidative stress markers, such as Fe^3+^ and MDA, decreased significantly when compared to the controls (Figure 2A,B, respectively).

Within this context, it is important to mention another study that also treated the RBC from healthy individuals with potential oxidizing agents, and later with HU. The authors observed that this would have an antioxidant potential since there was a decrease in lipid peroxidation and methemoglobin formation. The finding that HU could react preferably with the radicals formed by such oxidizing agents, would result in protection for the erythrocyte membrane [7]. This data is interesting because it would explain, at least in part, the beneficial effects resulting from the use of HU in patients with diseases, such as sickle cell anemia, and this drug could contribute to the reduction of free radicals. In 2012, a study by Torres et al. [35] reported a decrease in lipid peroxidation in the patients with sickle cell anemia who used HU when compared to the non-users. Another study that was developed by Brose et al. [36] reported that the treatment of rat neurons with neurotoxic agents, such as H_2_O_2_, and subsequently treated with HU, resulted in a reduction of the neurotoxic stress caused by these agents, as well as inducing an increase in the ATP content in these cells.

When relating the studies above to the current work, we realized that both of the concentrations of HU that were used for the treatment of the erythrocytes had, at first, an oxidizing effect. Over time, the erythrocyte protection mechanisms seem to be activated, thus reducing the damage to these cells. In diseases where there is natural oxidative stress, such as sickle cell anemia, the patients can be treated with HU, as the use of this drug can benefit those individuals affected by the disease because of its ability to reduce such stress. A study that was carried out with patients with sickle cell anemia, treated or not with HU, observed that the patients that were treated with the drug had a lower number of ROS and greater erythrocyte deformability when compared to the untreated patients [37]. The greater deformability of the RBC can be a favorable effect since such cells would present greater malleability and pass through the capillaries more easily. Despite the significant increase in hydrogen peroxide, the catalase levels did not show significant changes in any of the conditions that were evaluated in this study (Figure 3B and Figure 4B).

The enzymes that are responsible for removing endogenous H_2_O_2_ are peroxiredoxin II, an enzyme that is activated by concentrations of up to 0.41 mM of H_2_O_2_, and glutathione peroxidase, which can reduce H_2_O_2_, as well as the hydroperoxides and peroxynitrites [38]. In this study, the activity of the enzyme GPx was increased at both times of the treatment with HU, which may be related to the increase in H_2_O_2_ that was identified. Corroborating with the findings of this study, Choo et al. [39] demonstrated that a treatment with HU that was induced in the patients with sickle cell anemia, not only increased the GPx activity but also increased its expression.

The authors also observed that the GR activity increased at both of the treatment concentrations after 30 min (Figure 3D). This finding can be explained by an increase in the GPx activity in all of the samples treated with HU, which generated a greater amount of the substrate (GSSG), culminating in an increase in the activity of glutathione reductase, an enzyme that is responsible for maintaining the GSH levels in the RBC [40]. An increase in the GSH content resulting from the treatment of the two HU concentrations was observed in the current study after 30 min of incubation with the drug (Figure 3E). This finding is in line with the data reported by Teixeira and colleagues [41], who previously described an increase in the GSH that was induced by the treatment with HU in those patients with sickle cell anemia.

The activity of the G6PD and HEX enzymes was increased in both of the HU concentrations after 30 min of incubation. This data has suggested that the energy metabolism of the RBC was also stimulated by the presence of HU during the treatment times (Figure 5A,B). The PK activity was reduced at a concentration of 0.6 mM (Figure 5C), indicating that the pentose phosphate pathway was preferably more active under these conditions.

The fact that the pentose pathway was preferably active, instead of the glycolytic pathway, could explain the increase in Fe^3^+ that was found by the authors (Figure 1A) after the treatment with HU for 30 min. The NADH that was produced in this pathway was not available for use by the enzyme, cytochrome-b5 reductase, which is responsible for most of the interconnection of Fe^3^+ to Fe^2+^ (methemoglobin to hemoglobin) in the RBC.

The increased activity of HEX produced glucose-6-phosphate, a substrate for the G6PD enzyme, which plays an important role in the antioxidant system of the RBC since it produces NADPH [42], which is a cofactor for the production of GSH, one of the most important non-enzymatic antioxidants in the RBC. GSH was responsible for helping to protect the membranes against the superoxides, as well as other types of reactive oxygen species [40,43].

The induction of an increase in the G6PD activity was an important effect of HU since patients with sickle cell anemia have a restricted flow of NADPH. At low oxygen tensions, HbS binds very strongly to the cytoplasmic domain of band 3, a protein that is responsible for regulating glycolysis. Thus, the enzymes of the glycolytic pathway that are linked to the cytoplasmic domain of band 3 (phosphofructokinase and glyceraldehyde-3-phosphate dehydrogenase), remain in the cytoplasm, creating competition between the glycolytic and pentose pathways for the substrate (glucose- 6-phosphate), restricting the formation of NADPH [44]. In sickle cell anemia, the greater the release of the ROS, the stronger is the link between the cytoplasmic domain of band 3, HbS, and the hemicromes that are formed by the denaturation of hemoglobin. Increased G6PD activities enhance the production of NADPH and this might help to lessen the clinical effects caused by sickle cell anemia.

After the exposure of the RBC to HU for 1 h, a decrease in the GR activity was observed (Figure 4D) at the 0.6 and 0.8 mM HU concentrations, while there was an increase in the GSH content with 0.6 mM, followed by a decrease of this enzyme when using 0.8 mM of the drug (Figure 4E). In a previous study by this current group, a reduction of about 41% in the GSH content and 17% in the GR activity was observed in the blood samples from the patients with sickle cell anemia when treated with HU, compared to the patients who did not use this medicine [45].

These results might be explained by the reduction in the GSH levels and the activity of the enzyme G6PD, which was responsible for the production of NADPH (cofactor for GR) in the concentration of 0.8 mM HU; consequently, this would decrease the activity of the GR. In addition, the enzyme saturation might also explain the decreased activity of this enzyme. The G6PD might have had a decreased activity because there was no increase in the production of its substrate (glucose-6-phosphate) by the enzyme hexokinase, which remained without significant changes (Figure 6B) between the controls and those treated with HU.

Raththagala et al. [46] reported a significant increase in the ATP release in the HU-treated rabbit RBC after 20 min, due to the induction of greater RBC deformability. In these circumstances, the greater release of ATP could indicate a greater functioning of the enzymes in the glycolytic pathway. In this study, the authors did not find significant differences in the activity of the hexokinase enzyme after 1 h of incubation with 0.8 mM HU, although there was a reduction in the activity of pyruvate kinase at this concentration (Figure 6C). This data corroborates the possible inactivation of the glycolytic pathway under these conditions and that it was also reduced after the treatment with HU for 30 min. This has allowed the authors to infer that the treatments with HU most likely preferentially stimulated the pentose pathway and not the glycolytic pathway.

The Fe^3^+ content did not change significantly at the concentration of 0.6 mM HU when in relation to the controls, and it decreased when compared to the treatment with 0.8 mM HU. This result allows the authors to suggest that the increase in the activity of the enzyme, NADPH-methemoglobin reductase, helped in the process of converting Fe^3^+ to Fe^2+^ (methemoglobin to hemoglobin). The decrease in the content of Fe^3^+ might mean that the index of the reactive oxygen species decreased, as well as the oxidation of hemoglobin since a decrease in lipid peroxidation at a concentration of 0.8 mM was also identified.

When comparing the data that was obtained from the RBC that were treated with HU at concentrations of 0.6 and 0.8 mM after 1 h, it can be assumed that the latter concentration appeared to be more efficient in the fight against the free radicals. The experimental condition of the RBC that was incubated for 1 h with 0.8 mM HU was able to reduce the levels of the oxidative stress markers (hydrogen peroxide and lipid peroxidation—Figure 2B,C). It is also worth noting that 0.8 mM HU was the only dose capable of decreasing the Fe^3+^ content when compared with the control groups (Figure 2A).

Another interesting change when comparing both of the HU concentrations that were used in this study was the significant decrease in the SOD activity. This reduction, which was associated with the decrease in the concentration of Fe^3^+, allows the authors to infer that the oxidation process of hemoglobin decreased at the concentration of 0.8 mM HU after 1 h of incubation since Fe^3+^ and the superoxide anion (substrate for SOD) were products of the Hb oxidation [47,48].

It is important to highlight the difference that was identified between the HU concentrations that were used in this study. The activities of the HEX and G6PD enzymes (Figure 6A,B) and the GSH content (Figure 4E) decreased when the RBC were incubated with 0.8 mM when compared to 0.6 mM HU. Two hypotheses are to be considered: (1) the enzymatic saturation during the process of protection against the ROS. GR, which produces GSH, did not have the necessary cofactor (NADPH) produced by G6PD, which in turn required the production of glucose-6-phosphate by hexokinase to function; (2) the amount of the reactive oxygen species decreased in the presence of 0.8 mM of HU so that the production of GSH and NADPH was not stimulated, a fact that was confirmed by the decrease in lipid peroxidation (Figure 6B). The differences between the results obtained by the two HU concentrations (0.6 and 0.8 mM) have led the authors to reflect on what would be the best dose to be used by the patients with diseases of which HU is a part of the therapeutic scheme. In diseases, such as sickle cell anemia, in which the existence of oxidative stress is one of the main causes related to the clinical symptoms, the dose of 0.8 mM might have a better effect on the antioxidant system and the production of ROS; consequently, reducing the oxidative stress and therefore, benefiting the patients.

Although this study was carried out with the RBC from healthy individuals, the changes described in this study might help to elucidate the mechanisms of action of HU when administered for therapeutic purposes for certain diseases. The evidence in the literature indicates that the study’s results are similar to those reported in other studies that involve the treatment with HU, as in sickle cell anemia.

The effects of HU in those patients with sickle cell anemia go far beyond the increase in HbF and the decrease in cell production by the bone marrow. Further studies are still needed to understand other possible effects of using this drug on the RBC and whether these effects are harmful or not for the patient. The data presented in this paper raises the need for further investigations, to expand the effects of HU through other laboratory markers, opening perspectives for future studies. As an example, a more in-depth study of the HU effects on the composition and modulation of the RBC membranes can be suggested.

## Figures and Tables

**Table 1:** Comparison of the effects of HU at different concentrations and treatment times on the erythrocytes of healthy individuals when in relation to the control groups.

Time of Treatment	30 min		1 h	
HU Concentration	0.6 mM	0.8 mM	0.6 mM	0.8 mM
Variables				
Fe^3+^	↑	↑	-	↓
TBARS	-	↑	-	↓
H_2_O_2_	↑	↑	↑	↑
SOD	↑	↑	↑	↑
CAT	-	-	-	-
GPx	↑	↑	↑	↑
GR	↑	↑	↓	↓
GSH	↑	↑	↑	↓
G6PD	↑	↑	↑	↓
HEX	↑	↑	-	-
PK	↓	-	-	↓

TBARS = Thiobarbituric acid reactive substances, SOD = Superoxide dismutase; CAT = Catalase; GPx = Glutathione peroxidase; GR = Glutathione reductase; GSH = Reduced Glutathione; G6PD = Glucose-6-phosphate dehydrogenase; HEX = Hexokinase; PK = Pyruvate kinase; ↑ = a significant increase; ↓ = a significant decrease; - = no difference.

**Table 2:** Comparison of the data for the different markers when treating the erythrocytes with 0.8 mM versus 0.6 mM HU for 1 h.

Concentration	0.8 mM
Fe^3+^	-
TBARS	↓
H_2_O_2_	↓
SOD	↓
CAT	-
GPx	-
GR	-
GSH	↓
G6PD	↓
HEX	↓
PK	-

TBARS = Thiobarbituric acid reactive substances, SOD = Superoxide dismutase; CAT = Catalase; GPx = Glutathione peroxidase; GR = Glutathione reductase; GSH = Reduced Glutathione; G6PD = Glucose-6-phosphate dehydrogenase; HEX = Hexokinase; PK = Pyruvate kinase; ↑ = a significant increase; ↓ = a significant decrease; - = no difference.

## Data Availability

Data is contained within the article.
